# Comparative analysis of family consent to tissue donation according to two different donation form structures

**DOI:** 10.1590/S1679-45082014AO2555

**Published:** 2014

**Authors:** Manoela Gomes Grossi, Layse Beneli Prado, Geórgia Pereira Silveira Souza, Jaquelini Pereira dos Santos, Amanda Silva de Macêdo Bezerra, Cesar Augusto Guimarães Marcelino, Antônio Flávio Sanchez de Almeida, Andrea Cotait Ayoub

**Affiliations:** 1 Instituto Dante Pazzanese de Cardiologia, São Paulo, SP, Brazil

**Keywords:** Tissue donors, Tissue and organ procurement, Tissue transplantation, Brain death, Informed consent

## Abstract

**Objective:**

To define donors' profile of an Organ and Tissue Procurement Center and compare the family consent for tissue donation before and after modification of the Donation Term.

**Methods:**

A descriptive, documentary and quantitative study performed in an Organ and Tissue Procurement Center, analyzed 111 feasible donors' charts in the period from March 13 to September 13, 2010 (1st period), and from September 14, 2010 to March 14, 2011 (2nd period), based on the modification date.

**Results:**

The mean age of donors was 45.2 years, being 52.3% female. The causes of death included cerebral vascular accident (stroke) (64%), head trauma (27%), anoxic encephalopathy (2.7%), firearm injuries (2.7%) and others (3.6%). The notifications were predominantly of spontaneous origin (91%). Comparing the periods before and after the modification of the Donation Term, the donation consent for cornea increased by 17.2% and the consent for skin, bones, tendons and muscles had a discreet increase by 3.1%, 9.9% and 0.4%, respectively. On the other hand, there was decrease in consent for blood vessel (0.8%) and heart valves (4.1%) between the two periods.

**Conclusion:**

There was increase in family consent for donation of most tissues, but it was statistically significant only for cornea donation.

## RESUMO

**Objetivo:**

Traçar o perfil dos doadores viabilizados por um Serviço de Procura de Órgãos e Tecidos e comparar o consentimento familiar para doação de tecidos antes e após esta modificação do Termo de Doação.

**Método:**

Estudo descritivo, documental, quantitativo, realizado em um Serviço de Procura de Órgãos e Tecidos, analisou 111 prontuários de doadores viabilizados nos períodos de 13 de março a 13 de setembro de 2010 (1º Período) e de 14 de Setembro de 2010 a 14 de Março de 2011 (2º período), selecionados com base na data desta modificação.

**Resultado:**

Foi encontrada média de idade de 45,2 anos entre os doadores, sendo 52,3% do sexo feminino. As causas de morte incluem acidente vascular encefálico (64%), traumatismos cranioencefálicos (27%), encefalopatias anóxicas (2,7%), ferimentos por arma de fogo (2,7%) e outras (3,6%). As notificações foram predominantemente de origem espontânea (91%). Comparando os períodos antes e após a modificação do Termo de Doação, o consentimento para doação de córneas apresentou aumento de 17,2% e para doação de pele, ossos, tendões e músculos, discreto aumento de 3,1%, 9,9% e 0,4% respectivamente. Por sua vez, apresentou discreta diminuição o consentimento de vasos sanguíneos, de 0,8%, e valvas cardíacas de 4,1%, entre os períodos.

**Conclusão:**

Obtevese um aumento do consentimento familiar para a doação da maioria dos tecidos, porém com significância estatística apenas para doação de córneas.

## INTRODUCTION

Organ and tissue transplantation has evolved from an experimental treatment to an extremely effective therapeutic alternative to end-stage failure of some organs and tissues, leading to improvement in quality of life and life expectancy.^(1)^

According to the Brazilian legislation – Law No. 10.211/2001, tissues, organs and human body parts are made available to transplantation and other therapeutic purposes by family consent given by the spouse or relative over 18 years of age, respecting the succession line, straight or collateral, until the second degree kinship of the deceased donor. It is considered valid only if there is the signature of the Organ and Tissue Donation from Deceased Donor Form, as provided by the Transplantation Law No. 9434/1997.^(2–5)^

Effective tissue donor is the potential donor with diagnosis of brain death or arrested heart whose family consents with the donation.^(6)^ A potential donor is a patient whose first clinical test of the Brain Death Protocol was performed and is compatible with it, or someone with arrested heart, whose organs or tissues may be harvested for transplantation^(6)^.

Once such requirements are met, the family members are informed about the brain death of the patient and the family is interviewed by a trained professional, who instructs the family about the option of donating organs and tissues and tries to obtain their consent.^(1, 7)^

The interview intends to provide all pieces of information and the necessary support for the family to make decision about the donation.^(8)^ Among other issues, the family is infor med about the organs and tissues that may be donated; thus, family members may decide to donate or nor. If they agree, they have to choose the organs and tissues that will be donated by signing the Organ and Tissue Donation Form, which lists the possibilities of donation.

The decision made by the family to consent or refuse organ and tissue donation is influenced by a number of factors. A successful interview is related with family predisposition to donation, quality of the hospital care received, the moment in which the family hears about the possibility of donating the organs, privacy they have to discuss the topic and the skills and knowledge of the interviewer.^(1)^

Family refusal prevents the performance of transplantation, and, in 2008, the refusal rate in Brazil was 22.2%.^(4)^ Other potential barriers to transplantation are failure to identify and notify potential donors and clinical contraindications to donation.^(9)^

The main reasons for refusing donation reported by family members include: religion, lack of understanding or questions about the diagnosis of brain death, body handling, fear of family's reactions, inappropriate information and no confirmation of brain death, organ trade, inappropriate donation approach, patient's will, while alive, refusing to make the donation, fear of losing the loved one, and problems in care provided to the patient.^(1,4,10)^

The stages of the donation process converge to the family authorization and them the harvesting of the organs, carried out by the Organ and Tissue Procurement Center (SPOT, acronym in Portuguese), in the State of Sao Paulo, whose actions focus on obtaining organs and tissues from notified potential donors within the hospitals of a specific area.^(1, 5, 6)^

The Transplantation Center (CT) is part of the State Health Department of Sao Paulo, responsible for coordinating the State Transplantation System (SET),^(6)^ and a modification to the Donor Consent Term was made in September 2010.

Thus, the new donation term started to be used in interviews with family members, because it was believed that a modification to the term would favor an increase in tissue donation – the previous term used to make family members feel uneasy when deciding which organ or tissue to donate, as it was a check list that conveyed a derogative impression of the donation, as if it were a disassembling line, as reported by some families.

In the new consent term, the authorization to tissue and organ donation was set up in a descriptive fashion. The option ‘yes’ or ‘no’ for each organ and tissue was deleted from the form: now, it should be simply stated in writing which tissues or organ were chosen not to be donated.

The new Donation Consent Form started to be used in family interviews as of September 14, 2010, and it is still valid to present. Therefore, the objective was to define whether the modifications made to the term had already had impact on tissue donation.

## OBJECTIVE

To characterize the donors' profile from an Organ and Tissue Procurement Center in the period between March 2010 and March 2011, and to compare family consent for tissue donation before and after the modifications to the donation consent term.

## METHODS

A documental, retrospective, cross-section, quantitative study carried out in a SPOT of a public hospital specialized in cardiovascular diseases in the city of São Paulo.

**Figure 1:**
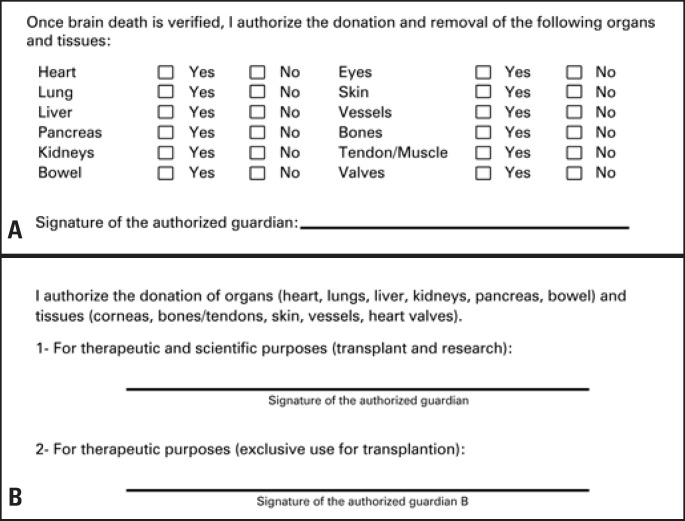
Models of Organ and Tissue Donation Consent Terms used before and after the modification

The charts of 111 donors identified by the center between March 2010 and March 2011 were analyzed for their characteristics. The charts were then divided into two independent groups – one between March 13 and September 13, 2010, and a second one between September 14 and March 14, 2011, considering the modification date of the Informed Consent. The “first period”, comprising the initial six months, represented the months before the term modification, and the “second period” were the six subsequent months, comparing family consent (Figure 1).

Donation Consent Term before (A) and after (B) modification.

Source: Secretaria de Estado da Saúde. Available from: http://www.saude.sp.gov.br/search.view?method=search&searchInAllField=true&searchType=2&search=true&searchFields=all%2Chtml%2CfileContent%2CfileName%2CmimeType&keywords=termodedoaçãodeórgãosetecidos&startPublicationDate=&endPublicationDate=&startObjectId=&page=1

The characterization of donors was made based on the variables gender, age, cause of death and type of notification – spontaneous, active search, hospital visit or telephone contact.

All notifications that received family consent for donation of organs and tissues were included, plus the donors who lost their donation capacity, despite the signed consent, due to heart arrest, positive serology and disposal of organs at poor status and absence of recipients.

The collected data were submitted to statistical analysis by Language and Environment for Statistical Computing, version 2011, by R Development Core Team.

The differences in proportions were calculated based on a 95% confidence interval. To check the association between the periods, the Fisher´s exact test was chosen as the most precise method for smaller samples and independent events, as it was observed during the period set by the authors.

The study project was approved by the Research Ethics Committee of the organization (Application No. 4171).

## RESULTS

There was slight predominance of females with a total of 58 female donors (52.3%) and 53 male donors (47.7%), mean age of 45.2 years.

**Figure 2:**
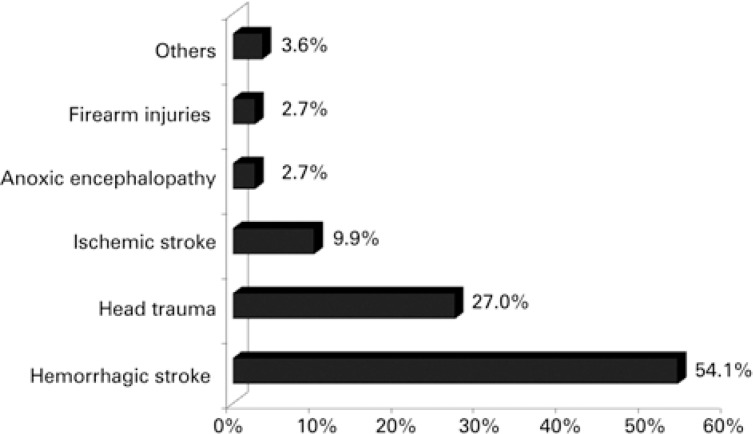
Main causes of brain death

The causes of brain death were: 60 (54.1%) hemorrhagic strokes; 30 (27%) head traumas; 11 (9.9%) ischemic strokes; 3 (2.7%) anoxic encephalopathy, 3 (2.7%) firearm wounds, and other causes including meningitis, embolism and subdural hemorrhage, amounting together to a total of 4 (3.6%) (Figure 2).

There was predominance of spontaneous notification in 101 cases (91%), active search by hospital visit was made in 6 cases (5.4%) and telephone search in 4 cases (3.6%). A total of 56.8% of consents were given in the first period and 43.2% were in the second period.

Family consent for cornea donations in the first period was 68.3%, whereas in the second period it was 85.4%, showing an increase in number of family consents. For skin donation, there were 31.7% consents given in the first period and 39.6% in the second one; for bone donation, 31.7% in the first period and 41.7% in the second one; for muscle and tendon donation, there were 41.3% in the first period and 41.7% in the second, characterizing a slight increase in the informed consent numbers in the second period.

For blood vessels, family consent was 50.8% in the first and 50% in the second period, and heart valves were donated in 93.7% and 89.6% in the first and second periods, respectively, showing slight reduction of tissue donation.

**Figure 3:**
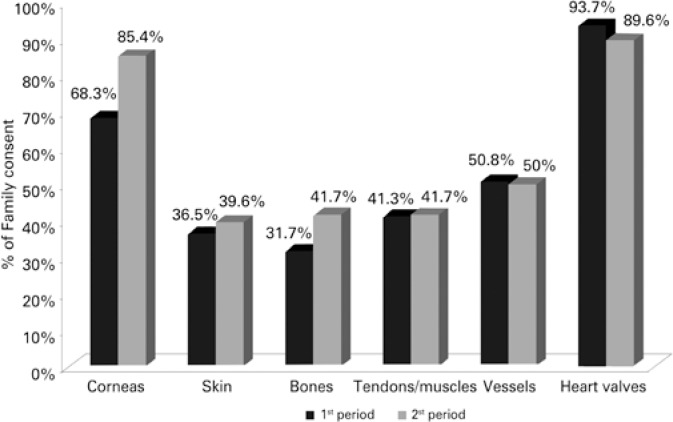
Family consent based on donated tissues

The informed consent for cornea donation has presented an increase by 17.2% from the first to the second period and there was slight increase by 3.1%, 9.9% and 0.4% for skin, bone and tendons and muscles, respectively. In turn, there was slight decrease in family consents for donation of blood vessels (0.8%) and heart valves (4.1%) between the two periods (Figure 3).

There was statistically significant improvement (p=0.0453) in the percentage of family consents for cornea donation in the second period. For the remaining tissues (skin, bones, tendons and muscles), the increase was not statistically significant. The same statement is valid for the reduction of family consent for donation of blood vessels and heart valves, which was not significant and could not be correlated with the modification of the consent term (Table 1).

**Table 1:** Proportional comparison of family consent to organ donation of the analyzed tissues

Tissues	Family acceptance		Difference %	p value***	CI (95%)	
	Before modification* %	After modification** %			LI**** %	LS***** %
Corneas	68.3	85.4	-17.2	0.0453	-34.2	-0.1
Skin	36.5	39.6	-3.1	0.8438	-23.2	17.0
Bones	31.7	41.7	-9.9	0.3216	-29.8	10.0
Tendons and muscles	41.3	41.7	-0.4	1.0000	-19.3	18.5
Vessels	50.8	50.0	0.8	1.0000	-18.8	20.4
Heart valves	93.7	89.6	4.1	0.4968	-8.3	16.4

* Family consents in the period before the modifications to the Donation Consent form;

** Family consents in the period after the modifications of Donation Consent form;

*** p value calculated by Fisher´s exact test;

**** Lower limit;

***** Upper limit; CI: Confidence interval.

## DISCUSSION

The profile of potential donors whose families have consented to organ and tissue donation identified by the present study is similar to that described in the literature.^(11, 12)^

Moraes et al.^(11)^ identified slight predominance of female donors and age range from 41 to 60 years. Concerning brain death causes, there was predominance of encephalic strokes, followed by head traumas and fewer cases of central nervous system tumors, firearm wounds, anoxic encephalopathy and meningites.

Medina-Pestana et al.^(11)^ reported mean age of donors of 50 years and the main causes of brain death were encephalic strokes, head traumas and firearm wounds. These results confirmed the findings of our present study, which are also confirmed by the study carried out by Cinque and Bianchi.^(13)^

Concerning type of notification, a study^(14)^ carried out in the same SPOT, in the period 1995 and 2008, with 241 donors identified the prevalence of 85% spontaneous notifications, showing that the same profile was maintained.

It was possible to infer that the structure of the Donation Consent Term is not directly related with the proportion of family consent to donation of tissues, reason why other strategies should be focused.

The investigation of family refusal is essential to support strategies that favor family consent to donation, in an attempt to minimize their obstacles to donation.

One aspect that greatly influences family decision is the previous formal manifestation of the deceased in favorable or against the donation, impacting family decision making. It frequently happens and Brazilian legislation states that organ donation is not assumed but rather consented, that is, regardless of the presence of documents or previous manifestations of the deceased, the family is owner of the consent or refusal to donation.^(2, 9)^

Moraes and Massarollo^(10)^ reported that the main reasons for family refusal to donation are: religious belief; not understanding the diagnosis of brain death and supposing there might be improvement in patient status; body handling; fear of family conflicts; inappropriate information provided by the clinical staff; fear of organ and tissue trade; patient's wish stated before death against organ donation, and fear of losing the loved one. These reasons - either direct or indirectly - may be related with decision making for tissue donation.

Dalbem and Caregnato^(4)^ identified as the main reasons for not approving organ and tissue donation as family lack of knowledge about the potential donor's wish to donate or not, formal manifestation against donation, family wish to keep the untouched body, and religious beliefs.

Absence of appropriate information by the clinical team to the family members generates distress and disappointment, which contributes negatively to refusal to donation consent.^(10)^

It is important to emphasize that brain death is often times a result from acute cerebral damage, which imposes an unexpected situation to the family, hindering family integrity which is essential when deciding for organ and tissue donation of a loved one.^(15)^

The support of the hospital organization and SPOT team to family members during the organ and tissue donation process is key to build a relationship of trust, eventually favoring the consent for donation.

Fusco et al.^(14)^ reported that the corneas were the most frequently donated tissues, whereas skin, bones, tendons and muscles and blood vessels were less donated, and they attributed this fact to lack of knowledge or difficulty in understanding by the population about the possibility of the donation, without causing any harm to the reconstitution of the donating body.

In our experience, the refusal of tissue donation is also related with fear of corpse mutilation.

The concern of the family with the integrity of the deceased body is combined with the belief that donation would dilacerate the corpse, which end up leading the family to say no to donation.^(16)^

The family interview should be carried out by a trained professional, because the performance and the skills of the interviewer may serve as a positive factor for donation, along with his/her empathy of the family feelings.^(7)^ The process of interviewing and gaining consent is essential for successful donation.

In addition to working on improving the factors that hinder donation, we should also focus on the facilitating factors, since the SPOT staff can effectively acts on them. Thus, the family interview becomes an enriching opportunity to these professionals, who should be familiar with the factors that favor this stage.

Santos and Massarollo^(17)^ reported that the factors that can facilitate family interview are: care provided not only to the potential donor, but also family support; clarifications about brain death; emotional status of the family; interview room and language used by the interviewer.

It is evident that some strategies should be focused on improving family interview techniques, in addition to carrying out studies about the barriers to family consent observed in each stage of the process of donation-transplantation, so as to favor family consent and availability of transplantation tissues.

The SPOT professionals should optimize the possibility of donation during the interview, making use of factors that influence family decision making and relying on previous experience and trained skills to aim at reaching a result that improves the whole process of donation.

Some limiting factors to the study were no data collected about the organs and family refusal rate, which may also have been influenced by changes to the Consent Term. There were a number of other data that could have influenced the decision for donating, but as they were not collected, they were not analyzed and compared; nevertheless, the authors are aware that it would have been extremely important and enriching to have had them analyzed as well.

## CONCLUSION

The study showed mean age of 45.2 years among donors and predominance of encephalic stroke as the main cause of death. The great majority of notifications were spontaneous.

There was statistically significant improvement in the percentage of family consents for cornea donation between the periods, which may be related with the modification to the donation consent term. The increase to donation consent observed for other tissues was not statistically significant.
